# Role of BRCA1-associated protein (*BRAP*) variant in childhood pulmonary arterial hypertension

**DOI:** 10.1371/journal.pone.0211450

**Published:** 2019-01-31

**Authors:** Ayako Chida-Nagai, Masaki Shintani, Hiroki Sato, Tomotaka Nakayama, Masaki Nii, Hiroyuki Akagawa, Toru Furukawa, Amer Rana, Yoshiyuki Furutani, Kei Inai, Shigeaki Nonoyama, Toshio Nakanishi

**Affiliations:** 1 Department of Pediatrics, National Defense Medical College, Tokorozawa, Saitama, Japan; 2 Department of Pediatric Cardiology, Tokyo Women’s Medical University, Shinjuku, Tokyo, Japan; 3 Department of Preventive Medicine and Public Health, Tokyo Medical University, Shinjuku, Tokyo, Japan; 4 Department of Pediatrics, Toho University Omori Medical Center, Ota, Tokyo, Japan; 5 Department of Cardiology, Shizuoka Children’s Hospital, Shizuoka, Shizuoka, Japan; 6 Institute for Integrated Medical Sciences, Tokyo Women’s Medical University, Shinjuku, Tokyo, Japan; 7 Department of Histopathology, Tohoku University Graduate School of Medicine, Sendai, Miyagi, Japan; 8 Division of Respiratory Medicine, Department of Medicine, University of Cambridge, Cambridge, United Kingdom; Kurume University School of Medicine, JAPAN

## Abstract

Although mutations in several genes have been reported in pulmonary arterial hypertension (PAH), most of PAH cases do not carry these mutations. This study aimed to identify a novel cause of PAH. To determine the disease-causing variants, direct sequencing and multiplex ligation-dependent probe amplification were performed to analyze 18 families with multiple affected family members with PAH. In one of the 18 families with PAH, no disease-causing variants were found in any of *BMPR2*, *ACVRL1*, *ENG*, *SMAD1/4/8*, *BMPR1B*, *NOTCH3*, *CAV1*, or *KCNK3*. In this family, a female proband and her paternal aunt developed PAH in their childhood. Whole-exome next-generation sequencing was performed in the 2 PAH patients and the proband’s healthy mother, and a BRCA1-associated protein (*BRAP*) gene variant, p.Arg554Leu, was identified in the 2 family members with PAH, but not in the proband’s mother without PAH. Functional analyses were performed using human pulmonary arterial smooth muscle cells (hPASMCs). Knockdown of *BRAP* via small interfering RNA in hPASMCs induced p53 signaling pathway activation and decreased cell proliferation. Overexpression of either wild-type BRAP or p.Arg554Leu-BRAP cDNA constructs caused cell death confounding these studies, however we observed higher levels of p53 signaling inactivation and hPASMC proliferation in cells expressing p.Arg554Leu-BRAP compared to wild-type BRAP. In addition, p.Arg554Leu-*BRAP* induced decreased apoptosis of hPASMCs compared with wild-type *BRAP*. In conclusion, we have identified a novel variant of *BRAP* in a Japanese family with PAH and our results suggest it could have a gain-of-function. This study sheds light on new mechanism of PAH pathogenesis.

## Introduction

Pulmonary arterial hypertension (PAH) is a progressive, potentially fatal disease[1]. Based on the Nice Classification in 2013[2], idiopathic PAH (IPAH) is a sporadic disease in which there is neither a family history of PAH nor an identified risk factor[3]. Heritable PAH (HPAH) is inherited in an autosomal dominant fashion with 10–20% penetrance[4].

In 2000, bone morphogenetic protein (BMP) receptor 2 (*BMPR2*) was identified as a primary gene for HPAH[5, 6]. *BMPR2* mutations have been identified in more than 70% of subjects with affected relatives and 11–40% of those with IPAH[3, 4].

Since then, other studies revealed further genes responsible for PAH. Mutations of the activin receptor-like kinase 1 gene (*ALK1*) were demonstrated in patients with hereditary hemorrhagic telangiectasia (HHT) associated with PAH[7]. Mutations in *endoglin* (*ENG*) have been more rarely identified in patients with PAH, predominantly with coexistent HHT[8–11]. We reported the first nonsense mutation of *SMAD8/9* in a PAH patient[12]. Moreover, we also reported 2 missense mutations of the bone morphogenetic protein receptor 1B (*BMPR1B*) gene and 2 missense mutations of *NOTCH3* gene in childhood PAH patients[13, 14]. Recently, caveolin-1 (*CAV1*) and *KCNK3* mutations were identified in PAH patients via whole-exome sequencing (WES) [15, 16]. Taken together, these genetic studies have increased our understanding of the molecular basis of PAH. However, more than half of PAH cases have no mutations in these genes.

This study aimed to identify a novel cause of PAH and clarify the pathogenesis of PAH using WES and human pulmonary arterial smooth muscle cells (hPASMCs).

## Materials and methods

**Fig 1:**
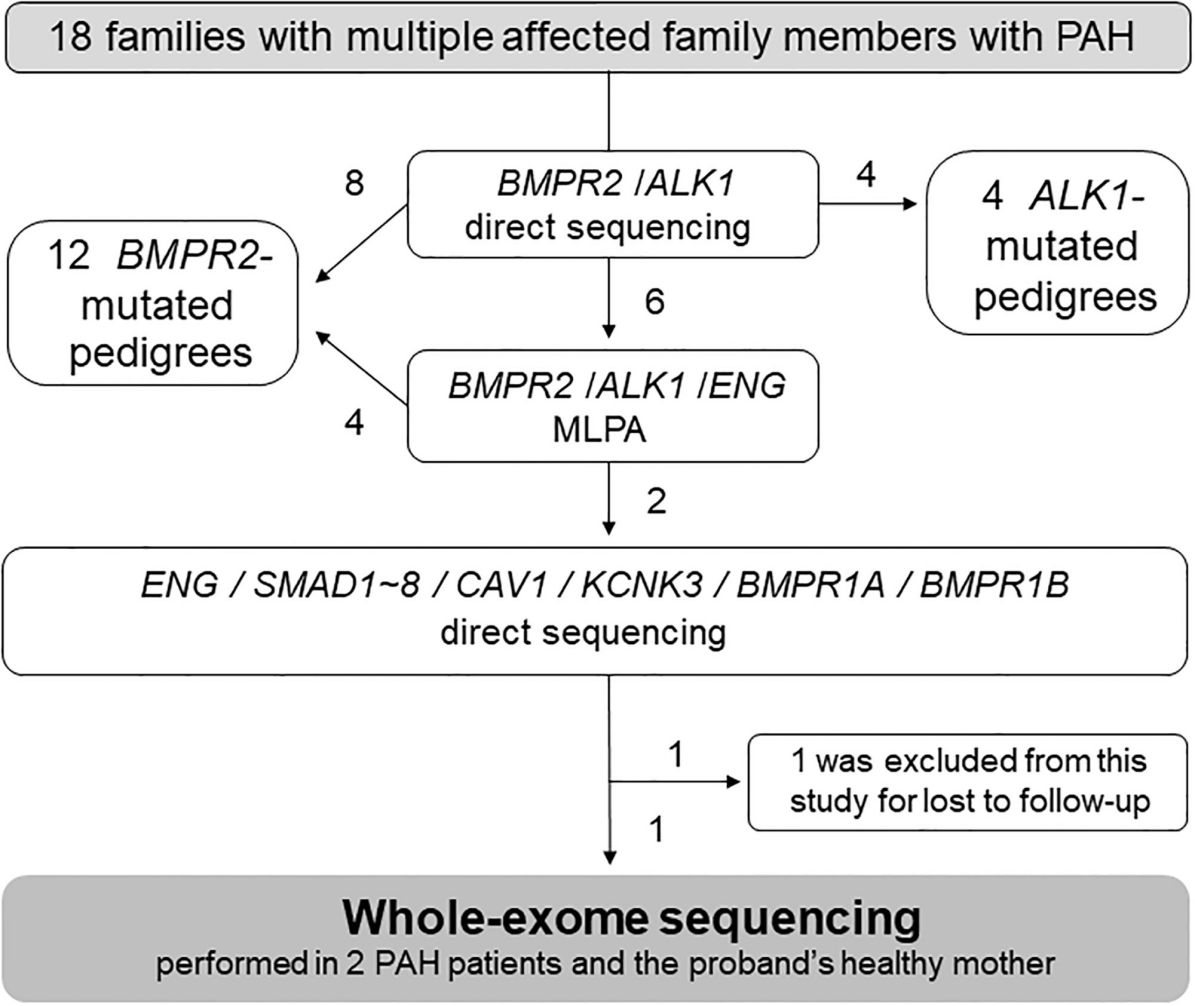
Patient disposition. PAH indicates pulmonary arterial hypertension; *BMPR2*, bone morphogenetic protein receptor type II; *ALK1*, activin receptor-like kinase 1; *ENG*, endoglin; *CAV1*, caveolin-1; *KCNK3*, potassium channel subfamily K member 3; *BMPR1A*, bone morphogenetic protein receptor type IA; *BMPR1B*, bone morphogenetic protein receptor type IB.

The study participants included 18 families with multiple affected family members with PAH (Fig 1).

Some of them were the same HPAH patients as in our previous study[12–14, 17]. The diagnosis of HPAH was made via a clinical evaluation, echocardiography, and cardiac catheterization based on the following criteria: mean pulmonary artery pressure of >25 mmHg at rest [18]. Patients with PAH associated with another disease such as portal hypertension and congenital heart disease were excluded from this study by trained cardiologists. This study was approved by the Institutional Review Committee of Tokyo Women’s Medical University (No. 313). Written informed consent was obtained from all patients or their guardians in accordance with the Declaration of Helsinki. When a pathogenic variant was detected, we confirmed that it was not present in 152 Japanese and 300 caucasian healthy controls by direct sequencing and also sequenced DNA samples from 109 IPAH patients to identify additional variants in the gene.

Genomic DNA was prepared from peripheral blood lymphocytes or lymphoblastoid cell lines transformed by the Epstein–Barr virus, as described previously[19]. A total of 12 *BMPR2*-mutated pedigrees and 4 *ALK1*-mutated pedigrees were detected using direct sequencing with an ABI 3130xl DNA Analyzer (Applied Biosystems, CA, USA) and multiplex ligation-dependent probe amplification (SALSA MLPA HHT/PPH1 probe set, MRC-Holland, Amsterdam, Netherlands). One of them was excluded from this study because the family was lost to follow-up. These 17 patients were excluded from this study (Fig 1). In one of the 18 families with PAH, no known disease-causing variants were found. In this family, a female proband and her paternal aunt developed PAH in their childhood.

In the family, WES was performed in the 2 PAH patients and the proband’s healthy mother. The extracted DNA was used for construction of a library using a SOLiD Fragment Library Construction Kit (Thermo Fisher Scientific). The library DNA was subjected to whole-exome enrichment using a TargetSeq Exome Enrichment Kit (Thermo Fisher Scientific). The enriched library DNA was then sequenced using the 5500xl SOLiD system, and was analyzed using Bioscope software (Thermo Fisher Scientific). Sequence data were mapped on Human Genome Reference, GRCh37/hg19 (The Genome Reference Consortium; http://www.ncbi.nlm.nih.gov/projects/genome/assembly/grc/index.shtml). All procedures were performed according to the manufacturers’ instructions. The WES results were filtered using dbSNP version 150 (https://www.ncbi.nlm.nih.gov/projects/SNP/), the 1000 genomes databases (Ensembl Release 91, http://asia.ensembl.org/index.html), Exome Variant Server version ESP6500 (EVS, http://evs.gs.washington.edu/EVS/), and the human genetic variation database version 2.3 (HGVD; Japanese genetic variation database, http://www.hgvd.genome.med.kyoto-u.ac.jp/) to remove polymorphisms and confirmed by direct sequencing with an ABI 3130xl DNA Analyzer (Applied Biosystems, CA, USA). Novel variants were analyzed for predicted effect on the protein using PolyPhen-2 (http://genetics.bwh.harvard.edu/pph2/), SIFT Human Protein database (Ensemble 63, http://sift.jcvi.org/www/SIFT_enst_submit.html), and CADD score version 1.3 (http://cadd.gs.washington.edu/). The functions of candidate genes were evaluated through Online Mendelian Inheritance in Man database (https://www.omim.org/) and the published literatures.

Human pcDNA3-GW-BRAP was kindly provided by Dr. Sylvie Urbe (Liverpool, UK). Site-directed mutagenesis was performed using a site-directed mutagenesis kit (Stratagene, CA, USA). The antibodies used were as follows: BRAP (Sigma, MO, USA), p53 (Cell Signaling Technology, MA, USA), phosphorylated p53 (Ser15, Cell Signaling Technology, MA, USA), p21 (Cell Signaling Technology, MA, USA), MDM2 (Santa Cruz Technology, TX, USA), BAX (BD Biosciences, NJ, USA), caspase3 (BD Biosciences, NJ, USA), NFκB (Cell Signaling Technology, MA, USA), BMPR2(Cell Signaling Technology, MA, USA), phosphorylated SMAD1/5/8 (Cell Signaling Technology, MA, USA), SF2/ASF (Cell Signaling Technology, MA, USA), and anti-β actin mouse antibody (Sigma, MO, USA).

As descrived in detail previously[20], distal hPASMCs were derived from the small vessels (<2 mm diameter) of lung resection specimens. All hPASMCs were harvested from the area enough far from each carcinoma. S1 Table shows the details of the patients from whom the cells were derived. No patients had pulmonary hypertension and received any chemotherapy and radiation therapy. The lung parenchyma was dissected away from a pulmonary arteriole, following the arteriolar tree, to isolate 0.5- to 2-mm-diameter vessels. These were dissected out and cut into small fragments, which were plated in T25 flasks and left to adhere for 2 h. A section of the pulmonary arteriole was collected, fixed in formalin, and embedded in paraffin, and sections were analyzed to ensure that the vessel was of pulmonary origin. The cells were maintained in DMEM/high-glucose (Sigma,St.Louis, MO) supplemented with 10% fetal bovine serum (Gibco, New York, NY) and 100units/ml penicillin-streptomycin (Gibco). The cells were incubated at 37°C in a humidified 5% CO_2_ incubator and used at passages 5–8. The use of the human tissues (Ethics Ref 08-H0304-56+5) was approved by Papworth Hospital ethical review committee, and informed consent was obtained from all subjects.

hPASMCs were transfected with small interfering RNA (siRNA) of the candidate gene (BRAPHS112138 for BRAP; Invitrogen, CA, USA), nontargeting siRNA (siCP; Dharmacon, CO, USA), or pcDNA3-GW-BRAP wild-type or variant, using DharmaFECT 1 transfection reagent (Dharmacon, CO, USA) for siRNAs or Lipofectamine 2000 reagent (Invitrogen, CA, USA) for plasmid. Transfection of siRNA was performed at a final concentration of 20 nmol/L. After an appropriate time for each experiment, hPASMCs were harvested and used for each analysis.

hPASMCs (1×10^4^) were incubated in 96-well plates for the indicated times for 10 days. Next, Guava EasyCyte flow cytometer was used to determine the number of proliferating cells and the percentage of mid-apoptotic cells. The values were analyzed using Guava ViaCount Software. Data were obtained from 4 independent experiments.

The viability of hPASMCs was determined by means of a cell proliferation assay using the WST-1 reagent (Roche, Basel, Switzerland). This assay is based on the cleavage of the tetrazolium salt WST-1 to formazan by cellular mitochondrial dehydrogenases.

For qRT-PCR, confluent cells were treated after siRNA or candidate gene plasmid transfection as described. The total RNA was prepared using the RNeasy Mini Kit (Qiagen, West Sussex, UK). DNase-digested total RNA (1000 ng) was reverse transcribed using SuperScript VILO Master Mix (Invitrogen) as described in the manufacturer’s instructions. Then, cDNA (30 ng/tube), PowerUp SYBR Green Master Mix (Applied Biosystems, CA, USA), and 200 nM of the relevant sense and antisense primers were mixed in a final volume of 10 μl, and the reactions were amplified on a 7500 Real-Time PCR System (Applied Biosystems). The primer details are shown in S2 Table. The relative expression of the target genes was normalized to β-actin using the ⊿⊿CT method and expressed as the fold change relative to the relevant control.

For nuclear and cytoplasmic fractionations, hPASMC lysates were prepared using the NE-PER fractionation kit (Thermo Fisher Scientific, Rockford, IL, USA) at specified time points, according to the manufacturer’s instructions. As described in detail previously[13], for western blotting, the lysates were separated on 10 to 20% resolving SDS-polyacrylamide gels, and proteins were transferred to polyvinylidene fluoride membranes via semidry blotting. The membranes were blocked in TBS-T (50 mM Tris-HCl [pH 7.6], 137 mM NaCl, 0.1% [w/v] Tween 20) containing 5% bovine serum albumin (BSA) or 5% skimmed milk. The membranes were rinsed with TBS-T and incubated with the primary antibody against BRAP (1:1000), p53 (1:1000), phosphorylated p53 (Ser15, 1:1000), p21 (1:1000), MDM2 (1:250), BAX (1:1000), caspase3 (1:1000), NFκB (1:1000), and β-actin (1:200,000). The membranes were rinsed with TBS-T and incubated with HRP-goat anti-rabbit IgG (Invitrogen) for BRAP, p53, phosphorylated p53, p21, BAX, caspase3, Bcl-2, and NFκB detection or with anti-mouse IgG (Invitrogen) for MDM2 and β-actin detection. Blots were then washed with TBS-T, and bound complexes were detected using enhanced chemiluminescence (ImageQuant LAS 4000 Mini, GE Healthcare). For detection of BMPR2 and phosphorylated SMAD1/5/8, Solution A (1 M Tris-HCl [pH 8.0], 50 mM, 0.5 M EDTA 1 mM [pH 8.0], 5 M NaCl 120 mM, 0.25% NP-40, 1% Triton X-100) was used to lyse hPASMCs. The lysates (50μg of protein) were provided to SDS-PAGE and western blotting in the same manner as described above. The membranes were incubated with the primary antibody against BMPR2(1:1000)and phosphorylated SMAD1/5/8 (1:1000). In addition, for co-immunoprecipitation, hPASMCs transfected wild-type or variant pcDNA3-GW-BRAP construct were lysed in Solution A. The lysates (250 μg of protein) were incubated with Dynabeads Protein G (Invitrogen), BRAP antibody (1 μg), or rabbit IgG (1 μg) and immunoblotted with p53 or p21 antibody and then with Clean-Blot IP detection reagent HRP (Thermo Fisher Scientific).It was difficult to get enough amount of nuclear fraction protein and cytoplasmic fraction protein separately for immunoprecipitation because of cell death after transfection, so we used whole protein for the immunoprecipitation.

Annexin V detection was performed using Guava Nexin Assay Reagent and Guava EasyCyte flow cytometer, according to the manufacturer’s instructions. The data was analyzed using the Guava Nexin Software (Guava Technologies, Hayward, CA, USA).

TdT-mediated dUTP-biotin nick-end labeling (TUNEL) assay was performed using the Guava TUNEL Kit and Guava EasyCyte flow cytometer, as described in the manufacturer’s instructions. The data was analyzed using the Guava TUNEL Software (Guava Technologies, Hayward, CA, USA).

Data are presented as the mean with standard deviation (SD). Differences cell counting, cell viability, qRT-PCR, western blotting and apoptosis assay between the 3 groups (negative control, BRAP siRNA and siCP or wild-type BRAP, p.Arg554Leu-BRAP variant and negative control) were evaluated by one-way analysis of variance (ANOVA) followed by the Dunnett’s test or Tukey’s test. Values of p<0.05 were considered significant. All statistical analyses were performed using JMP Pro 12 (SAS Institute, Cary, NC).

## Results

**Fig 2:**
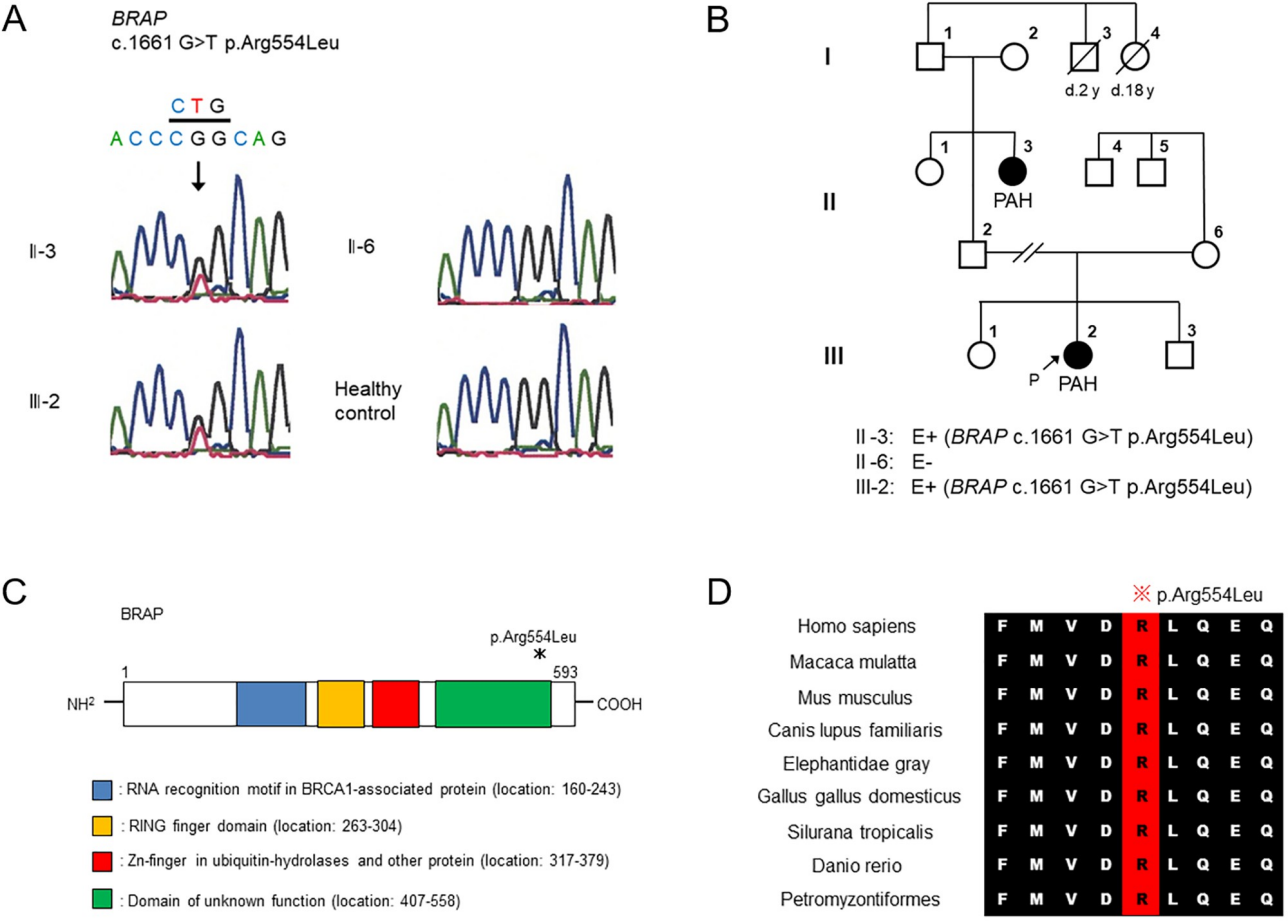
*BRAP* variant in a family with multiple affected family members with PAH. **A**, Pedigrees of the patients’ family. **B**, c.1661 G>T p.Arg554Leu was identified in ll-3 and lll-2. **C**, Schematic representation of the BRAP wild type and the location of the variant. **D**, Alignment of variant proteins among 9 species showing the conservation of arginine 554 in these species.

A *BRAP* variant (NM_006768.4, c.1661 G>T p.Arg554Leu) was identified in the 2 family members with PAH, which was absent in the proband’s mother without PAH (Fig 2A and 2B). The variant was not found in dbSNP, the 1000 genomes databases, the EVS, or the HGVD. It was regarded as “Probably damaging (0.998)” in the Polyphen-2 and “Damaging (0.01)” in the SIFT Human Protein. The CADD score was 35. In addition, it was absent in 152 Japanese healthy controls and 300 Caucasian healthy controls. Moreover, pathogenic variant of BRAP including p.Arg554Leu was absent in 109 IPAH patients who had no known disease-causing gene mutations.

Based on National Center for Biotechnology Information Reference Sequence (NP_006759.3, https://www.ncbi.nlm.nih.gov/), *BRAP* consists of a RNA recognition motif in BRCA1-associated protein, a RINGg finger domain, a Zn-finger in ubiquitin-hydrolases and other protein, and domain of unknown function (DUF). The p.Arg554Leu variant was located in DUF (Fig 2C). The alignment of the *BRAP* protein sequence between the 9 distantly related species showed that these amino acids were highly conserved (Fig 2D).

Clinical characteristics of the patients are mentioned as below. *Patient ll-3*: When she suffered from pneumonia at 3 months of age, cardiomegaly was pointed out, and atrial septum defect (ASD) was found in echocardiogram. Cardiac catheterization at 2 years of age already revealed severe pulmonary hypertension, that is, mean pulmonary arterial pressure (mPAP) of 85 mmHg and right atrial pressure (RAP) of 8 mmHg. Therefore, the advanced PAH could not be explained by ASD, she was diagnosed as IPAH with coincidental ASD. She spent uneventfully during childhood, but her oxygen saturation gradually declined and finally she presented so-called Eisenmenger physiology into adolescence. Epoprostenol infusion therapy and home oxygen therapy were started at the age of 20. After that, she maintained the World Health Organization (WHO) functional class III for 12 years, but eventually received lung transplantation and ASD closure concomitantly at the age of 34. *Patient lll-2*: When the patient was 2 years old, cardiac dilatation was identified in chest X-ray, and she was diagnosed with IPAH. Her hemodynamic data at 2 years of age revealed an mPAP of 65 mmHg, RAP of 5 mmHg, cardiac index of 3.92 l·min-1·m-2, and pulmonary artery wedge pressure of 7 mmHg. She has been receiving bosentan, sildenafil, and oral beraprost sodium. Her current condition is WHO functional class II at 11 years old. The patient’s hemodynamic data at 11 years of age revealed an mPAP of 62 mmHg, a RAP of 7 mmHg, a CI of 2.34 L·min^-1^·m^-2^, and a pulmonary artery wedge pressure of 12 mmHg. The patient’s current history includes partial epilepsy, borderline mental retardation, and delayed puberty. She has been receiving levetiracetam. Cases l-3 and l-4 died at 2 years old and 18 years old, respectively (Fig 2B). The cause of each death is unknown.

**Fig 3:**
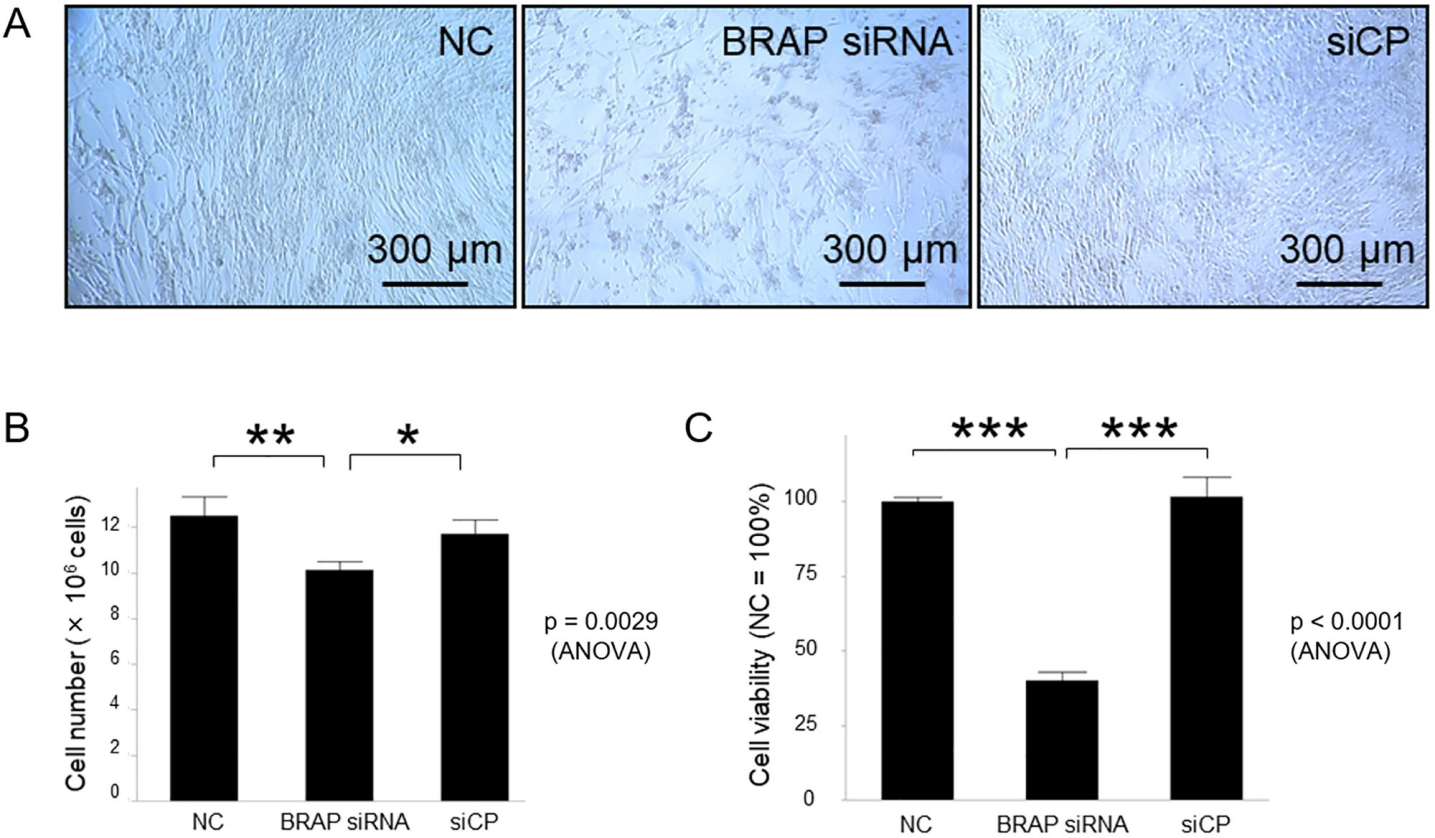
Knockdown of *BRAP* operates to decrease cell proliferation and cell viability in hPASMCs. **A**, hPASMCs growth 10 days after siRNA transfection. Scale bar, 300 μm. NC, negative control; siCP, nontargeting siRNA. **B**, Cell counting at 10 days after siRNA transfection. Each data point reveals the number of mean cells with standard deviation for four times (*p < 0.05, **p < 0.01). **C**, Cell viability was analyzed 10 days after siRNA transfection using the WST-1 reagent. The values represent the mean with standard deviation of 4 independent experiments and were converted to a percentage of the mean value relative to that of hPASMCs without any siRNA (***p < 0.001). The means were compared with that of BRAP siRNA using one-way ANOVA followed by Dunnett’s test.

We began by knocking down *BRAP mRNA* to assess its function in hPASMCs. Cell proliferation was assessed via Guava EasyCyte flow cytometer. *BRAP* siRNA in hPASMCs caused a decrease in cell number compared with the scrambled siCP and negative controls (Fig 3A and 3B). A cell viability assay was also performed using the WST-1 reagent. The hPASMCs transfected with *BRAP* siRNA showed lower levels of viability compared with that transfected with siCP and negative controls (Fig 3C).

**Fig 4:**
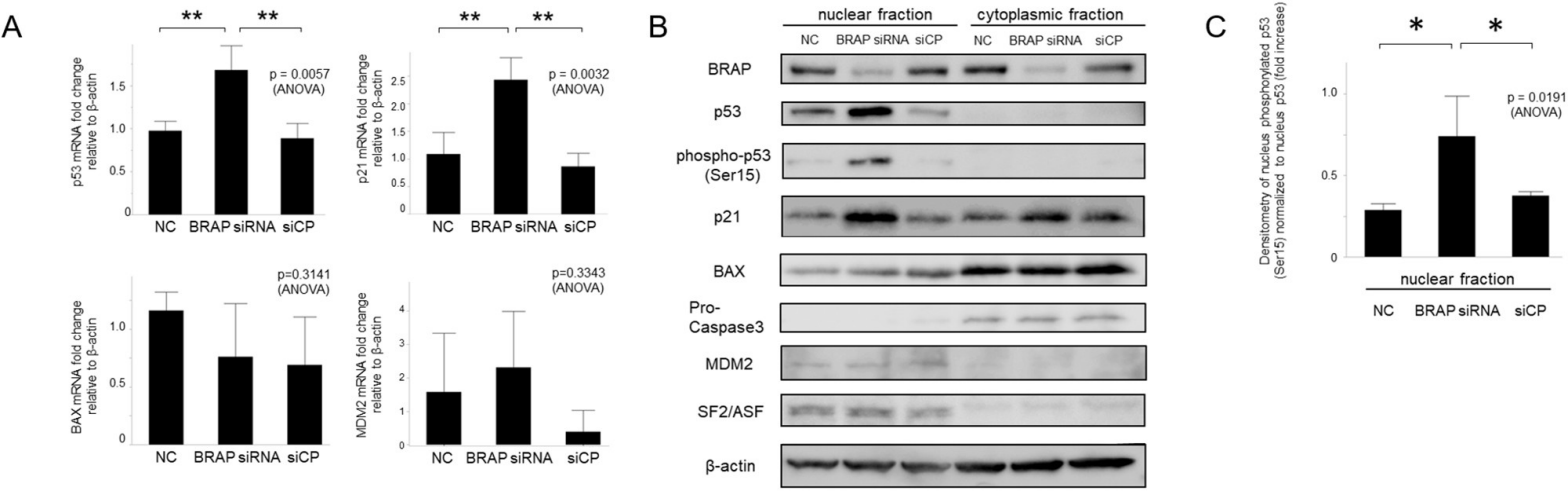
Knockdown of *BRAP* induced increasing p53 and p21 expression in the nucleus in hPASMCs. **A**, Relative abundance of mRNA transcripts for p53, p21, BAX, and MDM2 in hPASMCs. The total RNA was extracted 3 days after siRNA transfection. Expression was normalized to NC and represented as a fold change relative to β-actin (n = 3, *p < 0.05, **p < 0.01). NC, negative control; siCP, nontargeting siRNA. **B**, Western blotting of BRAP, p53, phosphorylated-p53, p21, BAX, pro-caspase3, and MDM2 expression. Cell lysates were collected 2 days after siRNA transfection in hPASMCs. The immunoblots also showed the level of siBRAP knockdown with SF2/ASF and β-actin as loading control. **C**, Extents of phosphorylation of p53 were shown relative to p53. Data represent mean values from 3 independent experiments. The means were compared with that of BRAP siRNA using one-way ANOVA followed by Dunnett’s test (*p < 0.05).

Next, qRT-PCR analysis was performed. The result showed that knockdown of *BRAP* in hPASMCs induces p53 and p21 expression in the nucleus (Fig 4A). As shown in Fig 4B, western blotting also revealed knockdown of *BRAP* in hPASMCs and induces higher expression of p53 and p21 in the nucleus. Phosphorylation of p53 in the nucleus was also increased significantly (Fig 4C).

**Fig 5:**
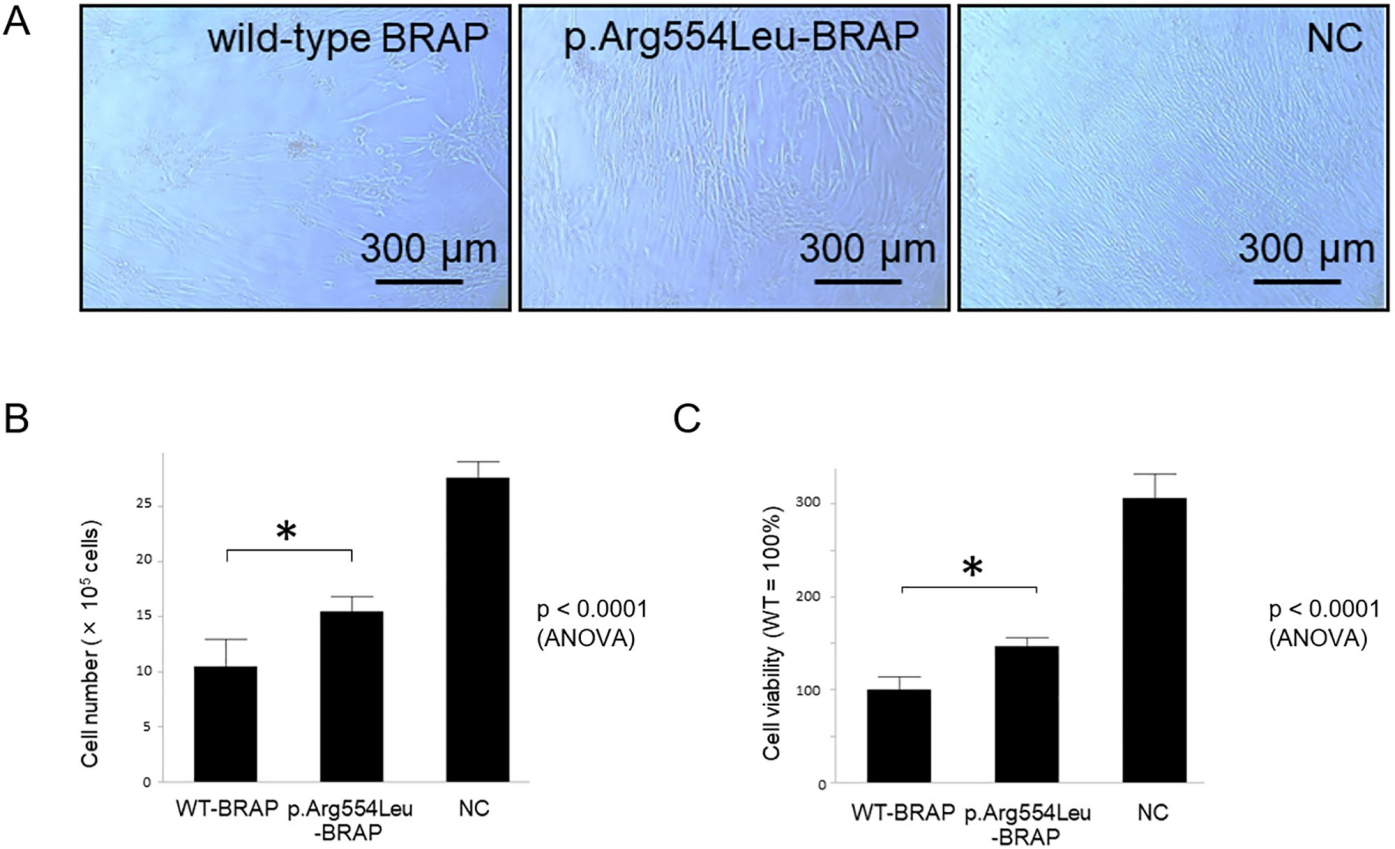
Overexpression of wild-type *BRAP* and p.Arg554Leu-*BRAP* variant decreased cell viability although this was less in cells expressing the p.Arg554Leu-*BRAP* variant. **A**, hPASMCs growth 10 days after wild-type *BRAP* or p.Arg554Leu-*BRAP* plasmid transfection. Scale bar, 300 μm. NC, negative control. **B**, Cell counting at 10 days after wild-type *BRAP* or p.Arg554Leu-*BRAP* plasmid transfection. Each data point reveals the number of mean cells with standard deviation for four times (*p < 0.05). WT-BRAP, wild-type *BRAP*; NC, negative control. **C**, Cell viability was analyzed 5 days after wild-type *BRAP* or p.Arg554Leu-*BRAP* plasmid transfection using the WST-1 reagent. The values represent the mean with standard deviation of 4 independent experiments and were converted to a percentage of the mean value relative to that of hPASMCs expressing wild-type BRAP (*p < 0.05).

Based on the findings of *BRAP* knockdown analysis, we investigated if the *BRAP* variant could influence cell proliferation and p53 signaling pathway. Overexpression of either wild-type or the p.Arg554Leu variant of BRAP caused a decrease in cell viability, confounding our analyses. Of note, cells expressing the p.Arg554Leu-BRAP variant did not exhibited as much of a decrease in viability and a relatively higher levels of hPASMCs proliferation compared to cells overexpressing wild-type BRAP(Fig 5A–5C).

**Fig 6:**
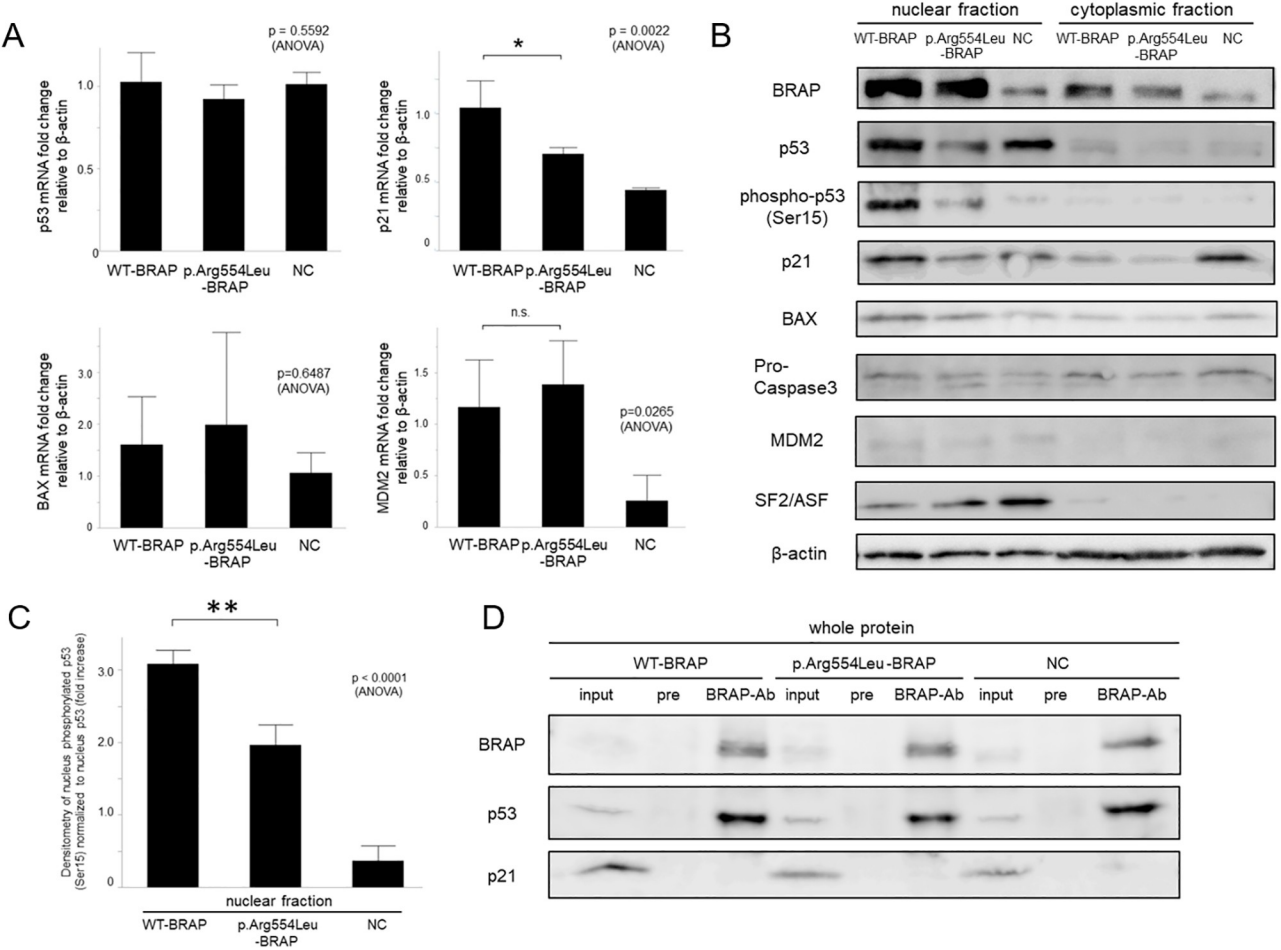
In cells expressing the p.Arg554Leu variant of BRAP, expression of p53, p21, and nuclear phosphorylated p53 is relatively lower compared to cells overexpressing wild-type BRAP. **A**, The expression of p53, p21, BAX, and MDM2 in hPASMCs was assessed using quantitative PCR. Total RNA was extracted 1 day after wild-type *BRAP* or p.Arg554Leu-*BRAP* plasmid transfection. Expression was normalized to NC and represented as a fold change relative to β-actin (n = 3, *p < 0.05). n.s., not significant; WT-BRAP, wild-type BRAP; NC, negative control. **B**, Effect of *BRAP* plasmid transfection on p53, phosphorylated-p53, p21, BAX, pro-caspase3, and MDM2 protein levels in hPASMCs by western blotting. Protein was collected 1 day after wild-type *BRAP* or p.Arg554Leu-*BRAP* plasmid transfection. The immunoblots also showed the effect of wild-type *BRAP* or p.Arg554Leu-*BRAP* plasmid transfection with SF2/ASF and β-actin as loading controls. **C**, The mean of the ratio of phosphorylated p53 to p53 densitometry was expressed. Data represent mean values from 3 independent experiments transfection (**p < 0.01). NC, negative control. **D**, Interaction between BRAP and p53. WT-BRAP, wild-type BRAP; pre, preimmune rabbit IgG; BRAP-Ab, anti-BRAP antibody.

qPCR revealed that compared with wild-type *BRAP*, p.Arg554Leu -*BRAP* variant in hPASMCs reduced p21 expression (Fig 6A). Western blotting showed the p.Arg554Leu -*BRAP* variant in hPASMCs induced a significantly lower expression of p53 and p21 and lower phosphorylation of p53 in the nucleus compared to wild-type *BRAP* (Fig 6B and 6C). As shown in Fig 6D, p53 co-immunoprecipitated with wild-type BRAP, p.Arg554Leu-BRAP or endogenous BRAP. Based on this result, it seemed that WT-BRAP might interacts with p53, although this result is not definitive and there is not significant difference between p53 expression levels induced by WT-BRAP and p.Arg554Leu-BRAP.

**Fig 7:**
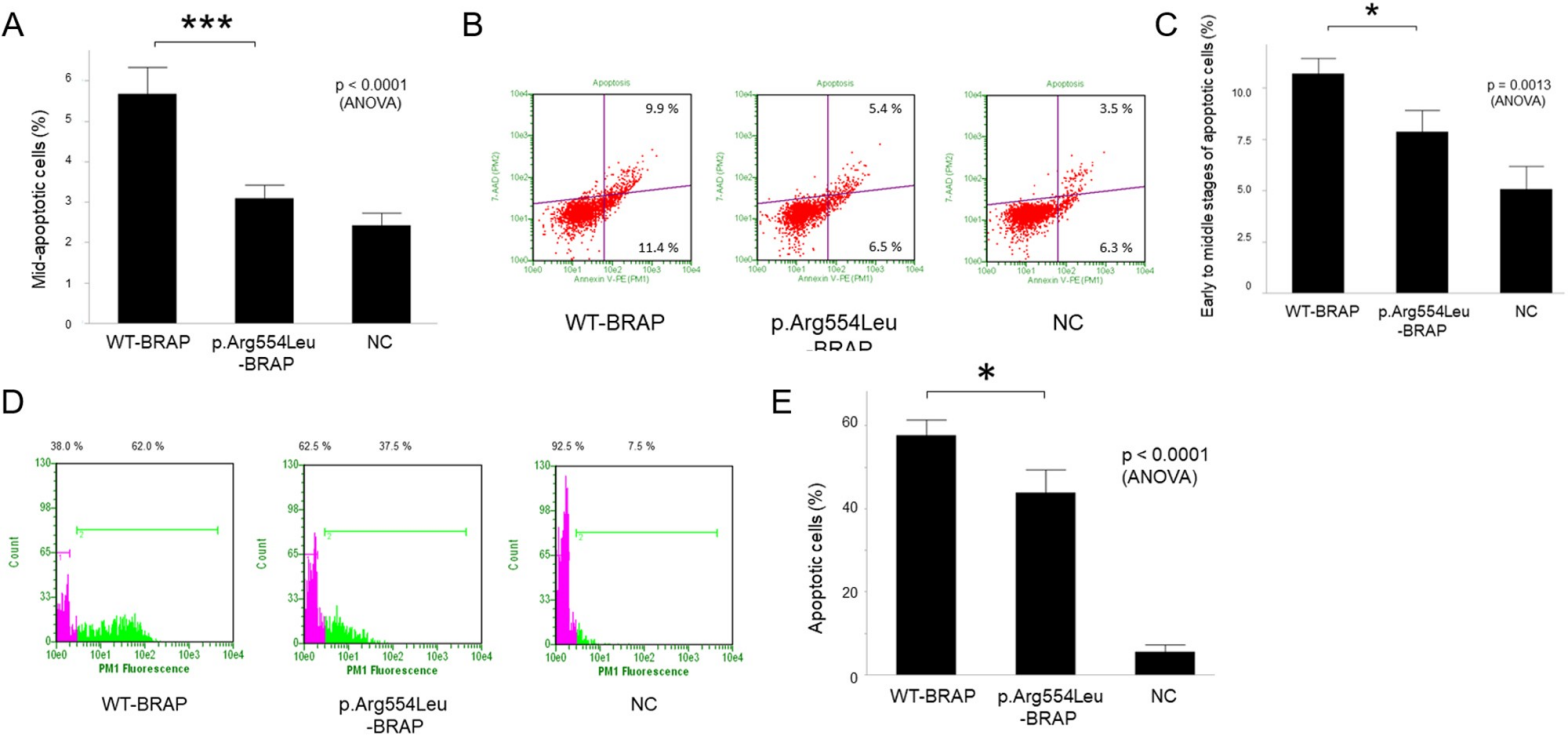
p.Arg554Leu-*BRAP* variant triggered decreasing apoptotic cells in hPASMCs. **A**, Apoptotic cells were determined using the Guava EasyCyte flow cytometer 10 days after wild-type *BRAP* or p.Arg554Leu-*BRAP* plasmid transfection preliminarily (n = 4, ***p <0.001). WT-BRAP, wild-type BRAP; NC, negative control. **B** and **C**, Annexin V assay was performed using the Guava EasyCyte flow cytometer 7 days after wild-type *BRAP* or p.Arg554Leu-*BRAP* plasmid transfection (n = 3, *p <0.05). **D** and **E**, Effect of *BRAP* plasmid transfection on hPASMCs apoptosis induced evaluated by TUNEL assays. TUNEL staining cells were determined using Guava EasyCyte flow cytometer 3 days after wild-type *BRAP* or p.Arg554Leu-*BRAP* plasmid transfection (n = 3, *p <0.05).

In a preliminary experiment using the Guava EasyCyte flow cytometer, it was observed that compared with wild-type *BRAP*, the p.Arg554Leu -*BRAP* variant induced a decrease in apoptotic cells in hPASMCs (Fig 7A). Next, we performed an Annexin V assay. As shown in Fig 7B and 7C, the percentage of early- to mid-apoptosis following transfection of p.Arg554Leu -*BRAP* plasmid was significantly lower than in hPASMCs transfected with wild-type *BRAP* plasmid. In addition, TUNEL assay was also conducted and showed that the BRAP variant increased the rate of TUNEL-positive staining cells (Fig 7D and 7E).

## Discussion

This is the first study to report on a *BRAP* variant in a family with multiple PAH patients. In addition, we also described originally that BRAP could affect the growth and apoptosis of hPASMCs through p53 signaling pathway.

*BRAP* is located on chromosome 12q24 and has been shown to interact with nuclear localization signals[21]. Based on the Human Protein Atlas version 18 (https://www.proteinatlas.org/), BRAP is predominantly expressed in the testis and distributed to several organs including the lung and smooth muscle. Here we show that BRAP is clearly expressed in hPASMCs (Fig 4), demonstrating that BRAP function may be important in this cell type.

In our study, knockdown of *BRAP* induced decreasing cell proliferation in hPASMCs. On the other hand, expression of wild-type and the p.Arg554Leu variant of BRAP caused a decrease in cell viability/proliferation compared to the negative control of hPASMCs transfected with empty pcDNA3-GW. This confounded our overexpression analyses and their comparison to our knock-down analyses. However, overexpression of an oncogene the causes a decrease in cell viablilty is not unique, as the same has been observed with activated RAS [22]. The role of BRAP in cell proliferation may depend on the availability of sufficient quantities of molecular partners and BRAP may not be able to cause increases in cell proliferation when overexpressed by itself in the conditions used in our in vitro experiment. Moreover the apparent toxicity of both WT-BRAP and p.Arg554Leu-BRAP when overexpressed compared to the negative control, might be down to domains within BRAP or p.Arg554Leu-BRAP negatively interacting with survival factors in the cell when they are expressed at artificially high levels in our system. Because of this confounding issue, rather than comparing the effect of overexpressing WT-BRAP and p.Arg554Leu-BRAP to the negative control, we also compared the effect of overexpressing WT-BRAP and p.Arg554Leu-BRAP directly with each other. We accept this choice has limitations but that it is also reasonable because the transfection condition is the same in these two samples. Under these conditions, one interpretation of our results could suggest that the overexpression of p.Arg554Leu variant of BRAP has a relatively less negative effect on cell survival (ie it has relatively higher proliferation and decreased apoptosis), or perhaps a more protective effect on viability, compared to wild-type BRAP overexpression.

On balance, our functional analyses could suggest that the variant had a gain-of-function effect on hPASMCs. It is known that PAH is induced by abnormal pulmonary vascular remodeling based on increasing hPASMCs proliferation and decreasing hPASMCs apoptosis causing obstruction of small peripheral pulmonary arteries based on abnormal growth of hPASMCs[23], thus our study could contribute to the elucidation of PAH pathogenesis. This said, unfortunately due to limited availability of human PASMCs we were unable to purse these experiments further to allow us to provide a definitive understanding to the functional role of the mutation.

Recently, BRAP has attracted attention because its expression is associated with the prognosis of patients with colorectal cancer [24] and the invasiveness of esophageal squamous cell carcinoma[25]. In addtion, several studies revealed that BRAP is genetic risk factor for coronary artery disease including myocardial infarction, hypertension, metabolic syndrome[26] [27] [28] [29] [30]. In these studies, 5 single nucleotide polymorphisms (SNPs) were reported (rs11066001[chr12:111681367, c.443+270 A>G], rs3782886 [chr12:11672685, c.90 A>G, p.Arg241Arg)], rs11065987 [chr12:111634620, located 9.9 kb upstream of *BRAP*], rs1544396 [chr:111625071, located 17 kb upstream of *BRAP*] and rs601663 [chr:111685480, c.82+231 T>C]), however all SNPs are located very far from the variant we identified, c.1661 G>T p.Arg554Leu. Thus, to understand the role of BRAP in PAH more study of how the p.Arg554Leu mutation affects BRAP function are needed. For example this might include using gene editing to introduce a p.Arg554Leu knock-in into a relevant cell type.

Some reports demonstrated the relationship between BRAP and NFκB, one of the coordinating regulators of inflammation. Ozaki *et al*. found that BRAP knockdown by siRNA suppressed activation of NFκB on coronary endothelial cells[27]. Zhao *et al*. reported that *BRAP* knockdown induced a dramatic decrease in nuclear translocation of NFκB and overexpressed BRAP protein caused an increase in nuclear NFκB translocation in esophageal squamous cell carcinoma[25]. In addition, Liao *et al*. demonstrated that BRAP could enhance NFκB nuclear translocation in human aortic smooth muscle cells[31]. However, our study showed that both *BRAP* knockdown and p.Arg554Leu-BRAP variant did not affect NFκB localization (S1 and S2 Figs). These differences may be caused by the different types of cells used in the various studies. Based on our study, it seemed that the effect of p.Arg554Leu-*BRAP* variant on NFκB signaling pathway is potentially less relevant than the p53 signaling pathway on hPASMCs, although our conclusion is limited due to the problems associated with decreased cell viability in our overexpression studies.

We also investigated whether *BRAP* knockdown or p.Arg554Leu-*BRAP* plasmid transfection could affect BMP signaling in which there were several disease-causing genes in PAH[5–13], however neither influenced the expression of BMPR2 or phosphorylation of SMAD1/5/8 (S3 Fig). Again, this analysis was confounded by the problems we encountered with our overexpression studies.

After the analysis of *BRAP* variant effect for NFκB and BMP signaling, we decided to analyze the p53 pathway. The p53 pathway plays various roles: control of cell proliferation, apoptosis, senescence and adaptation to metabolic or oxidative stress, and so on[32]. Previous studies have connected the p53 signaling pathway to PAH pathogenesis. Activated signaling of p53 tumor suppressor protein was suggested to induce prevention and reverse experimental pulmonary hypertension. Jacquin *et al*. demonstrated that inactivation of p53 is sufficient to induce development of pulmonary hypertension in rats[33]. Mouraret *et al*. also showed that p53 activation induced by Nutlin-3a prevents and reverses experimental pulmonary hypertension[34]. In addition, it was reported that p53 gene deficiency promotes hypoxia-induced pulmonary hypertension[35]. Furthermore, Piccolo *et al*. revealed that p21 in the nucleus inhibits cell proliferation by blocking cyclin-dependent kinases[36]. Although the exact function of BRAP in hPASMCs is still unclear, the results of our study are very compatible with these previous reports. According to the report by Mouraret et al^27^, slight changes in p53, p21 and phosphorylated p53 protein expression can significantly affect senescence in hPASMCs. This finding further supports that the p.Arg554Leu variant in BRAP might be a gain-of-function and contribute to the pathogenesis of PAH.

Our data suggested that WT-BRAP interacts with p53. Although it appears that p.Arg554Leu-BRAP may also precipitate with p53 in this experiment, we cannot say this definitely due to the low expression levels of WT-BRAP and p.Arg554Leu-BRAP and because the BRAP antibody we use picks up both endogenous and exogenously overexpressed WT and the BRAP variant. However, our data at least show that the expression of p.Arg554Leu-BRAP does not increase the expression level of p53 as much as WT-BRAP does.

Asada *et al*. reported that in monocytes, BRAP interacts directly with p21 *in vitro* and *in vivo*, based on immunoprecipitation analysis[37]. We tested this interaction in hPASMCs between BRAP and p21 by immunoprecipitation study but we did not obtain the same result (Fig 6D).

In addition, BRAP is known to have E3 ligase activity and regulates MAP kinase activation negatively[38]. It is reported that SMAD-specific E3 ubiquitin protein ligase 1, SMURF1 is increased in patients with PAH and is critical to the development of experimental PAH[39]. Thus, the role of BRAP as an E3 ligase might be important for PAH pathogenesis. Further investigation is required to explore this hypothesis.

From the clinical information, the 2 PAH patients with the *BRAP* variant in the same family were diagnosed in their early childhood and seemed to have a similar severity of phenotype. The father of proband (ll-2) should have the same *BRAP* variant, but he has no clinical signs of PAH to date. The reasons of the discrepancy in the phenotype are unknown, but may be related to the difference in sex or may be due to the low penetrance. Further investigations are necessary.

In addition to the limitation of our study stated above, we identified several other limitations of our current study. We found a *BRAP* variant in only one family with multiple affected family members with PAH, and we could not perform gene analysis of the II-2 in the family. In addition, we could not perform gene analysis for the other family members. Therefore, an investigation in a larger number of PAH subjects is mandatory. We performed BRAP overexpression and siRNA knock-down experiments only in hPASMCs. To understand the physiological function of BRAP and its role in other cardiovascular cells cells, further analyses are necessary. Moreover, we could not separate exogenous BRAP expression by plasmid transfection to endogenous BRAP because of lack of hPASMCs. Furthermore, we could not clarify how BRAP works with the p53 signaling pathway in hPASMCs concretely. The precise mechanisms how *BRAP* variant causes PAH still remain unclear. Additional analysis using human pulmonary arterial endothelial cells (hPAECs), PAH patients-derived hPASMCs/hPAECs and animal models with the *BRAP* variant is necessary to further analyze the function of BRAP in the pathogenesis of PAH.

## Supporting information

S1 Table Pulmonary arterial smooth muscle cell clinical information. (DOC)

S2 Table Primer sequences for quantitative PCR. (DOC)

S1 Fig Western blotting of NFκB expression after BRAP knockdown in hPASMCs. Protein was collected 2 days after siRNA transfection. The figure of BRAP, SF2/ASF,and β-actin bands is same as Fig 4B.
(TIF)

S2 Fig No effect of BRAP plasmid transfection was observed on NFκB expression and localization in hPASMCs by western blotting. Protein was collected 1 day after wild-type BRAP or p.Arg554Leu-BRAP plasmid transfection.
(TIF)

S3 Fig BRAP knockdown or p.Arg554Leu-BRAP plasmid transfection did not influence BMPR2 expression and phosphorylation of SMAD1/5/8 in hPASMCs by western blotting. Whole protein was collected 2 days after siRNA transfection or 1 day after wild-type BRAP or p.Arg554Leu-BRAP plasmid transfection.
(TIF)
