# Supplementary material for: Role of BRCA1-associated protein (BRAP) variant in childhood pulmonary arterial hypertension
Source: PLoS One. 2019 Jan 31;14(1):e0211450. doi: 10.1371/journal.pone.0211450 (PMC6355015; doi:10.1371/journal.pone.0211450)
Supplement: S1 Table — (DOC) [file pone.0211450.s001.doc]

| Subject ID | Sex | Age | *BMPR2* mutation | Clinical details |
| --- | --- | --- | --- | --- |
| 77MP | Female | 64 y | None | Adenocarcinoma |
| 79MP | Male | 60 y | None | Squamous cell carcinoma |
| 85MP | Female | 64 y | None | Squamous cell carcinoma |
