# Supplementary material for: Role of BRCA1-associated protein (BRAP) variant in childhood pulmonary arterial hypertension
Source: PLoS One. 2019 Jan 31;14(1):e0211450. doi: 10.1371/journal.pone.0211450 (PMC6355015; doi:10.1371/journal.pone.0211450)
Supplement: S2 Table — (DOC) [file pone.0211450.s002.doc]

| Target | Forward primer | Reverse primer |
| --- | --- | --- |
| Human  β-actin | 5’-GCACCACACCTTCTACAATGA-3’ | 5’-GTCATCTTCTCGCGGTTGGC-3’ |
| Human  BRAP | 5’-AAAGGGCTGAAGTGCTGAATC-3’ | 5’-TCTGGCGTTTGACAGTATCGGC-3’ |
| Human  p53 | 5’-ACCACCATCCACTACAACTACAT-3’ | 5’-ACAAACACGCACCTCAAAGC-3’ |
| Human  p21 | 5’-CAGATCCACAGCGATATCCA-3’ | 5’-ACGGGACCGAAGAGACAAC-3’ |
| Human  BAX | 5’-CACGGCAGAGAATGCCTATGA-3’ | 5’-CCCAATTGATGCCACTCTCAA-3’ |
| Human  MDM2 | 5’-ACCTCACAGATTCCAGCTTCG-3’ | 5’-TTTCATAGTATAAGTGTCTTTTT-3’ |
