# Supplementary figures and images for: Role of BRCA1-associated protein (BRAP) variant in childhood pulmonary arterial hypertension
Source: PLoS One. 2019 Jan 31;14(1):e0211450. doi: 10.1371/journal.pone.0211450 (PMC6355015; doi:10.1371/journal.pone.0211450)

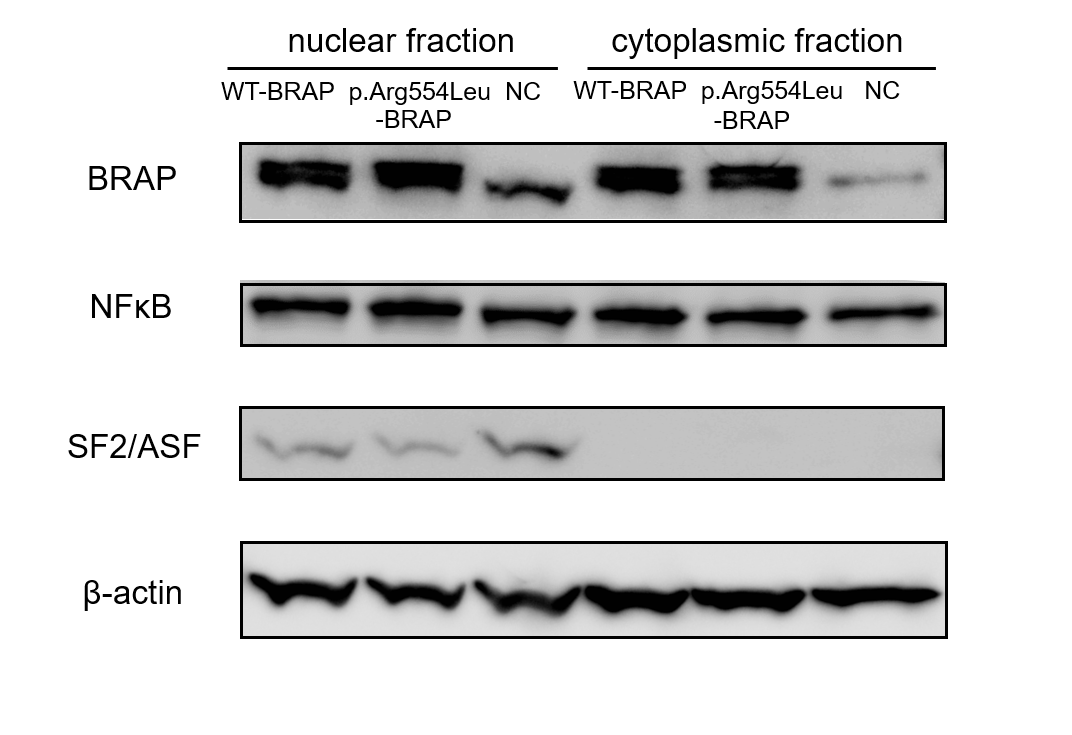

Supplement: S1 Fig — Protein was collected 2 days after siRNA transfection. The figure of BRAP, SF2/ASF,and β-actin bands is same as Fig 4B. (TIF) [file pone.0211450.s003.tif]

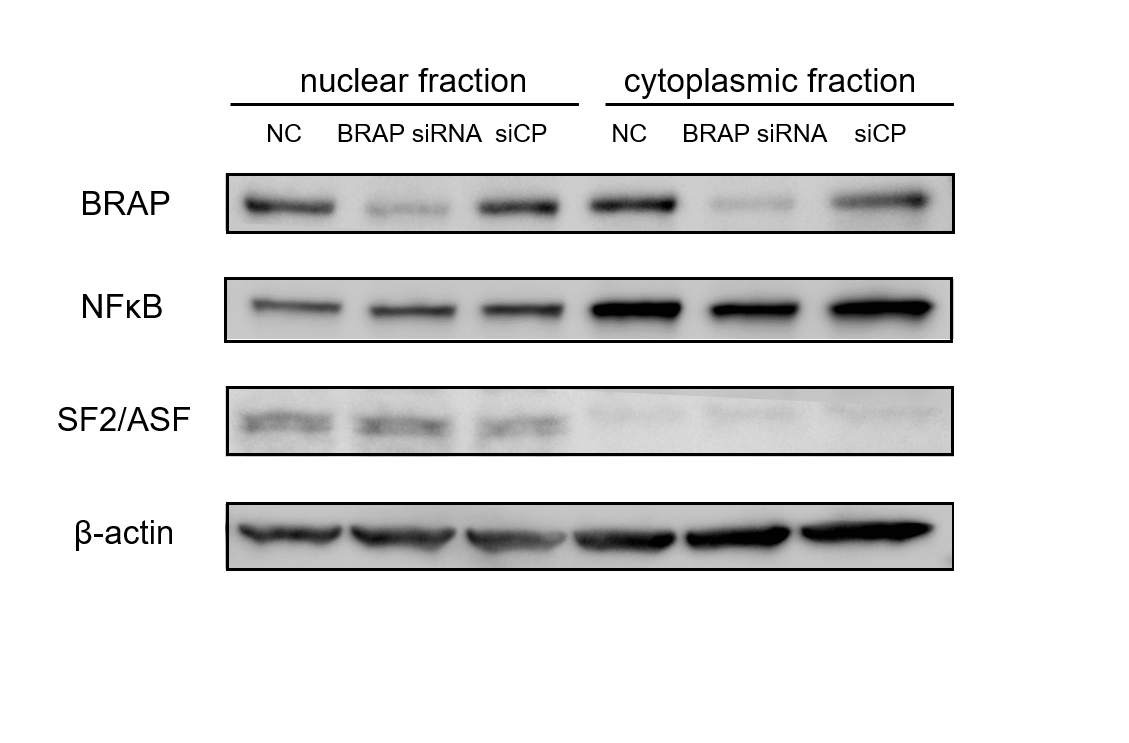

Supplement: S2 Fig — Protein was collected 1 day after wild-type BRAP or p.Arg554Leu-BRAP plasmid transfection. (TIF) [file pone.0211450.s004.tif]

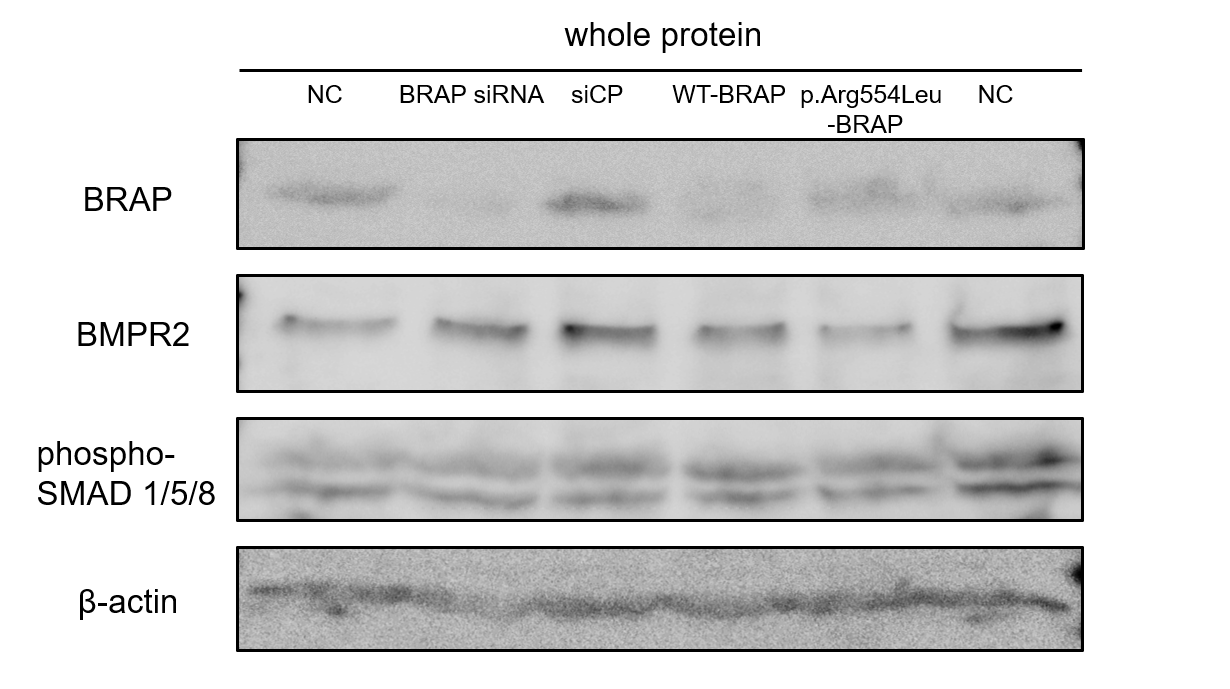

Supplement: S3 Fig — Whole protein was collected 2 days after siRNA transfection or 1 day after wild-type BRAP or p.Arg554Leu-BRAP plasmid transfection. (TIF) [file pone.0211450.s005.tif]
